# Strategies for streamlining uterine topographic classification in placenta accreta spectrum

**DOI:** 10.1016/j.xagr.2026.100636

**Published:** 2026-03-26

**Authors:** Sarah P. Walker, Helena C. Bartels, Albaro J. Nieto-Calvache, José M. Palacios-Jaraquemada, Sally L. Collins, Rozi Aryananda

**Affiliations:** 1 Birmingham Women’s and Children’s NHS Foundation Trust, Birmingham, UK (Walker and Collins); 2 Dept of UCD Obstetrics and Gynaecology, School of Medicine, University College Dublin, National Maternity Hospital, Dublin, Ireland (Bartels); 3 Departamento de Ginecología y obstetricia, Fundación Valle del Lili, Cali, Colombia (Nieto-Calvache); 4 Programa de Ginecologia y Obstetricia, Facultad de Salud, Universidad del Valle, Cali, Colombia (Nieto-Calvache); 5 Department of Obstetrics and Gynecology, CEMIC University Hospital, Buenos Aires, Argentina (Palacios-Jaraquemada); 6 First Anatomy Chair, School of Medicine, University of Buenos Aires, Buenos Aires, Argentina (Palacios-Jaraquemada); 7 Nuffield Department of Women’s and Reproductive Health, University of Oxford, John Radcliffe Hospital, Oxford, UK (Collins); 8 Department of Obstetrics & Gynecology, Faculty of Medicine Universitas Airlangga, Dr Soetomo General Academic Hospital, Surabaya, Indonesia (Aryananda); 9 Department of Obstetrics & Gynecology, Erasmus University Medical Center, Rotterdam, The Netherlands (Aryananda)

**Keywords:** one-step conservative surgery, placenta accreta, uterine dehiscence, uterine topographic classification, uterine vasculature

## Abstract

Effective management of anterior placenta previa, uterine dehiscence, and placenta accreta spectrum (PAS) requires a comprehensive understanding of uterine topography and vascular anatomy, knowledge that typically develops with time and through experience. This article aims to present a simplified, practical framework to support clinicians in PAS referral centers managing only a few cases per year, offering practical strategies to overcome challenges frequently encountered during the incorporation of individualized, topography-based surgical management. Recognizing anatomical patterns, anticipating complications based on topographical type, and tailoring vascular control strategies to each case allows for more confident, uterine-sparing surgical approaches when appropriate, and timely transition to hysterectomy when required. While most cases will fall within the more manageable upper peritoneal reflection placenta accreta types, clinicians must be prepared for the occasional complex or rare presentation. Ongoing collaboration between institutions, humility in seeking support, and integration of telecompanionship can help ensure that these rare cases are handled with the benefit of collective expertise.

## Introduction

A detailed topographical understanding of the uterus, surrounding spaces, and vascular anatomy has become an essential tool for improving the management of placenta accreta spectrum (PAS) and uterine dehiscence.^1^ For the operating surgeon, grasping the anatomical rationale behind hemostatic techniques and suture placement is critical for intraoperative decision-making and optimal patient outcomes.

**AJOG Global Reports at a Glance**

**Why was this study conducted?**

To provide a simplified, practical framework for clinicians in PAS referral centers who manage a limited number of cases and may have less experiential understanding of the topographical classification system and its role in surgical planning and patient outcomes.

**Key findings**

Recognition of uterine topographical patterns and vascular anatomy enables anticipation of surgical challenges, supports confident selection of uterine-sparing techniques when appropriate, and facilitates timely transition to hysterectomy when required.

**What does this add to what is known?**

This article translates complex anatomical and surgical principles into an accessible, reproducible approach that supports decision-making in lower-volume PAS centers. It promotes appropriately selected conservative surgery and has the potential to help reduce maternal morbidity.

Developing this expertise takes time and experience. PAS is best managed in specialized referral centers with appropriate multidisciplinary expertise and resources. In high-volume regions such as parts of Latin America, Southeast Asia, and North Africa, frequent case exposure, allows clinicians to develop a nuanced appreciation of uterine topography and its clinical implications.^2^, ^3^, ^4^ In contrast, many clinicians worldwide practice in lower-volume centers, where suggested benchmarks for excellence, such as managing a minimum of 2 cases per month are unrealistic.^5^ In regions, such as parts of Africa, Western and Central Asia, Europe and Oceania, annual case-load remains below 10.^3^^,^^6^ This challenge is compounded by contemporary training structures that expose obstetricians to progressively less open abdominal surgery, leaving many poorly prepared for complex cesareans.^7^, ^8^, ^9^ Given the increasing rates of complex cesarean sections, including multiple repeat cesareans, previa accreta, and obesity, this is a concerning trend.

Women with suspected PAS should be referred to the highest level of expertise available within regional or national healthcare systems. However, in some countries, low birth rates and high vaginal delivery rates mean that even tertiary or national PAS referral centers manage relatively few cases annually, making further centralization impractical. In such settings, care is commonly delivered in multidisciplinary tertiary hospitals with limited case exposure, highlighting the need for structured frameworks to support safe decision-making, including appropriate selection between conservative approaches and planned cesarean-hysterectomy. In lower-volume centers, reduced team familiarity with uterine-sparing techniques means that planned cesarean hysterectomy may represent the safest option, particularly in emergency situations. This is supported by a survey from 22 international society of PAS centers across 16 countries, in which fewer than half performed uterus-preserving procedures, and only two centers managed more than five such cases during this 30-month timeframe.^10^ Barriers for not attempting focal resection were limited experience and concerns regarding increased blood loss.^10^

Although evidence suggests adopting the topographical classification and use of the one-step resective-reconstructive technique reduces hysterectomy rates, their primary value in lower-volume settings lies in improving preoperative understanding of PAS heterogeneity, anticipating operative complexity and hemorrhage risk, thereby guiding the surgical decision-making rather than promoting conservative surgery as a default.^11^ With appropriate training, multidisciplinary support, and increasing experience, some centers may selectively and progressively introduce uterine-sparing approaches. Given the clinical heterogeneity of PAS, centers of excellence should be equipped to offer a range of management options to ensure optimal, individualized care.

Emerging evidence supports reduced surgical morbidity with resection-reconstructive surgery compared with cesarean hysterectomy,^11^^,^^12^ including lower blood loss, fewer genitourinary injuries, and reduced need for intensive care admission in a meta-analysis of 2300 women.^13^ In addition, hysterectomy carries a significant psychological burden, particularly due to the loss of fertility.^14^ However, outcomes are highly dependent on local expertise and available resources, signifying the importance of centers auditing their own outcomes. Further large, multicenter, long-term studies evaluating both short- and long-term sequelae are needed before definitive conclusions can be drawn.

This article presents a simplified, practical framework for understanding uterine topography and vascular anatomy to guide individualized surgical management across centers with varying case volumes. While recognizing that planned hysterectomy may remain the safest option in many low-volume settings, the framework aims to improve risk assessment, surgical planning, and safety, and to provide a foundation for centers to build confidence, refine technique, improve safety, and where appropriate, minimize surgical invasiveness in carefully selected cases.

## Placenta previa, non-PAS uterine dehiscence, and PAS

Placenta previa, non-PAS uterine dehiscence, and PAS represent a continuum of conditions related to placental implantation within the lower uterine segment (LUS), all associated with increased risk of postpartum hemorrhage.^15^ In placenta previa, bleeding stems from weaker LUS contractility compared to the fundus.^16^ In the context of a previous cesarean scar, the risk is further amplified by the reduction of functional myometrium or the addition of fibrosis, dense adhesions, and neovascularization associated with PAS.^17^^,^^18^

Many PAS cases are now recognized to occur alongside areas of non-PAS uterine dehiscence, where extreme thinning of the myometrium allows placental herniation into the uterine serosa.^19^^,^^20^ While PAS and uterine dehiscence frequently coexist, they can also present independently.^21^ Differentiating between the two conditions can be difficult, even for experts, since their ultrasonographic and intraoperative appearances are similar.^4^

Nonetheless, the surgical principles for managing isolated uterine dehiscence with placenta previa are often the same as those used in PAS due to the similarly compromised uterine wall. In both cases, the lack of a contractile, healthy myometrium substantially increases the risk of hemorrhage if it is not appropriately addressed.^11^

Prenatal ultrasound helps to determine the likelihood of uterine scar dehiscence with an underlying nonadherent placenta from high-grade PAS.^13^ It also plays a critical role in guiding clinical suspicion and ensuring timely referral to regional centers of excellence equipped to manage such cases. However, a definitive diagnosis can only be confirmed following surgical staging and clinical assessment to determine of whether the placenta can be separated from the underlying uterine interface.^22^ To improve surgical management and outcomes in cases of LUS remodeling with previa, the topographical classification was proposed.

## Topographical classification

Topographic classification defines PAS according to which uterine wall is affected (anterior, lateral, or posterior), the vertical level of the lesions (above or below the vesicouterine peritoneal reflection), and the nature of the lesions (characterized by the presence of neovascularization or dense adhesions).^23^ This approach creates a conceptual 3D map of the uterus, guiding tailored management options; one-step conservative surgery (OSCS), total hysterectomy, or modified subtotal hysterectomy (MSTH).^1^^,^^24^ In contrast to the International Federation of Gynecology and Obstetrics (FIGO) classification, which emphasizes depth of invasion and histopathological features, the topographic approach is designed to inform operative strategy and individualized surgical management.

**Figure 1:**
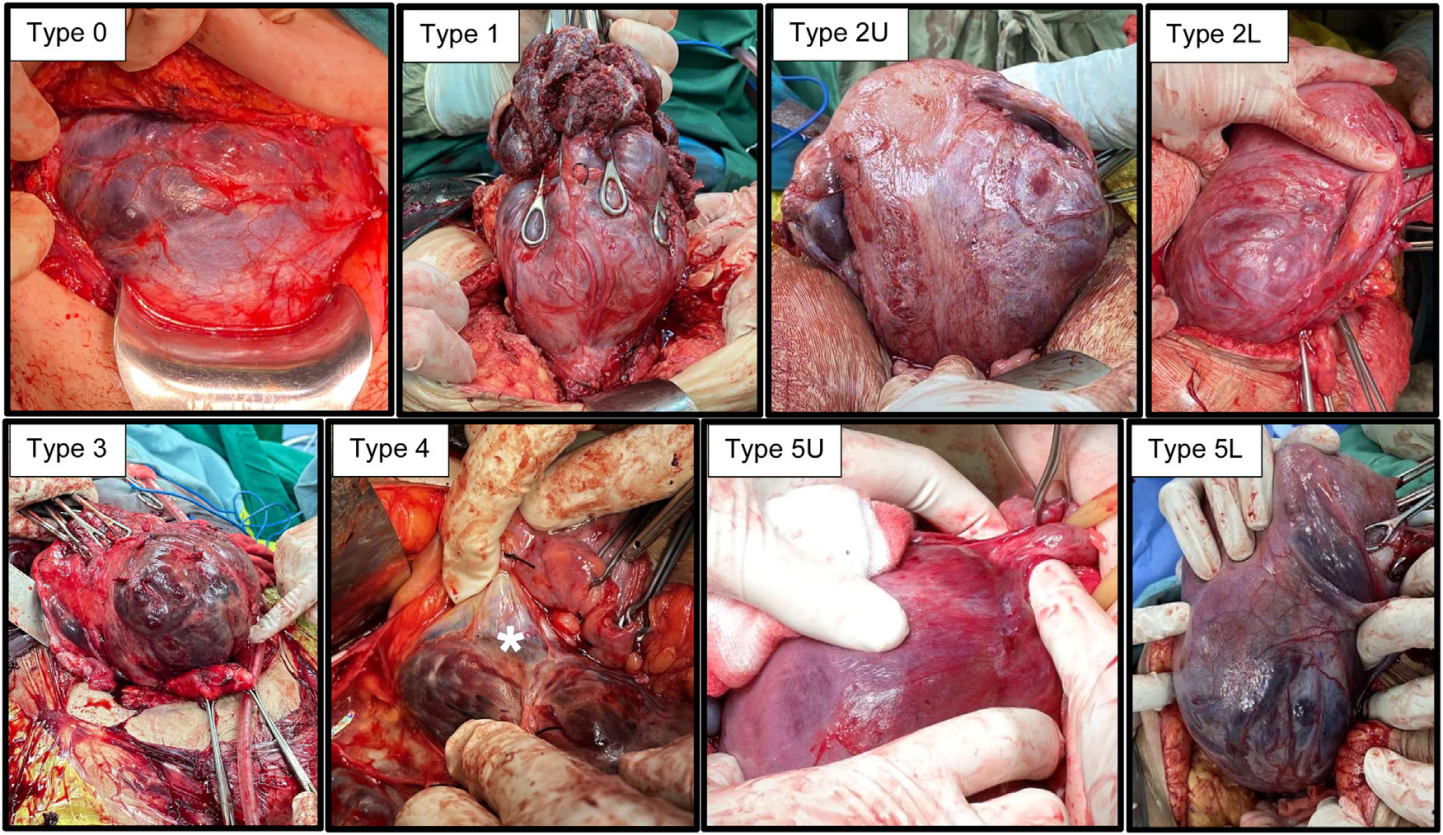
Topographical grades of PAS. Above are surgical pictures of the intraoperative findings in the eight different types of PAS *Dense adhesions between the bladder and the lower uterine segment made separation of the two organs impossible.

The topographical classification system comprises eight primary types, with final classification determined intraoperatively following surgical staging (Table and Figure 1). Type 0 refers to uterine dehiscence with placenta previa, while Type 1 involves the anterior upper uterine wall. Type 2 denotes lateral uterine wall involvement and is subdivided into 2U (upper) and 2L (lower). Type 3 includes participation of the anterior lower uterine wall with or without cervical involvement. Type 4 represents Type 3 disease with associated vesicouterine dense adhesions. Type 5 represents posterior uterine wall involvement and is further subdivided into 5U (upper) and 5L (lower).^1^

**Table:** Topographic classification for placenta accreta spectrum, alongside their associated blood supply, and recommended treatment strategies

**Grade**		**Description of location**	**Comprehensive surgical staging (nature of lesion)**	**The most probable source of blood supply**	**Vascular control strategy recommended**	**Surgical treatment proposed**
**Type 0**		Uterine dehiscence is limited to the **anterior** upper or lower uterine segment (sectors S1 and/or S2). The lesion’s appearance closely resembles that of type 1, and the differential diagnosis can be made only when attempting to separate the abnormal myometrial segment from the underlying placenta	**Minimal neovascularization, dissecting plane present**	Colpouterine arteries Uterine arteries	Ligation of CUA	OSCS
**Type 1**		Involvement of the upper portion of the **anterior** uterine segment within sector S1 (with or without upper vesicouterine adherence)	**(A) No external evidence** **(B) Neovascularization** **(C) No dissecting plane/fibrosis**	Vesicouterine pedicles Colpouterine arteries Uterine arteries	Ligation of VUP and CUA	OSCS
**Type 2**	**Upper**	Lateral uterine wall involvement: above the level of the peritoneal vesicouterine reflection, bulging to the upper part of the parametrium (sector S1)	On parametrial opening: **(A) No external evidence** **(B) Purple tissue and bulge or neovascularization** **(C) B+neovascularization**	Uterine artery Colpouterine artery NFV (branches of the Obturator artery, the Internal Iliac artery)	Ligation of VUP and CUA Unilateral ligation of the uterine artery	OSCS
	**Lower**	Bulging to the lower part of the parametrium, the lower limit of the bulge is not evident after opening the broad ligament during surgical staging (sector S2)		NFV (branches of Ureteric artery, internal iliac artery, cervical artery, vaginal artery) Colpouterine artery	IRAA occlusion Additionally, it is advisable to apply a ureteral stent to safely dissect the ureter before proceeding with the hysterectomy	Total hysterectomy (IRAA)
**Type 3**		Bulge reaching the lower uterine segment below the peritoneal vesicouterine reflection, or the cervix (sector S2)	**(A) No external evidence** **(B) Neovascularization** **(C) No dissecting plane**	Vesicouterine pedicles Colpouterine arteries Uterine arteries	Ligation of VUP and CUA	OSCS Total hysterectomy
**Type 4**		Type 3 PAS **PLUS** vesicouterine adherence in the lower uterine segment, near the cervix	**It is impossible to find a dissecting plane due to extensive fibrosis**	Vesicouterine pedicles Colpouterine arteries Uterine arteries	Ligation of the VUP up to the area where bladder dissection can be achieved Infrarenal abdominal aortic occlusion (IRAA) during subtotal hysterectomy, up to the application of a compression suture on the uterine-cervical stump	Total hysterectomy MSTH (IRAA)
**Type 5**	**Upper**	**Posterior** uterine wall involvement confined to sector S1	On uterine exteriorization: **(A) No external evidence** **(B) Purple tissue or vascularization** **(C) Organ involvement**	Ovarian artery NFV (branches of Inferior mesenteric artery)	Ligature of identified neurovascular bundles (NFVs) Compression sutures in the myometrium surrounding the area to be resected	OSCS
	**Lower**	Bulging reaching lower uterine segment or cervix (sector S2)		NFV (branches of anterior rectal artery)	IRAA occlusion	Total hysterectomy (IRAA)

The middle column delineates surgical staging according to the nature of the lesion. The traffic-light color coding system highlights the levels of surgical difficulty; **green** = less surgical complexity, **brown** = lesions with intermediate surgical complexity, and **red** = lesions with high surgical complexity and a high risk of massive bleeding—adapted from Nieto-Calvache et al.^30^

*CUA*, colpouterine arteries; *IRAA*, infrarenal abdominal aorta; *MSTH*, modified subtotal hysterectomy; *NFV*, newly formed vessels; *OSCS*, one-step conservative surgery; *VUP*, vesicouterine pedicles.

*Walker. Strategies for streamlining uterine topographic classification in placenta accreta spectrum. AJOG Glob Rep 2026.*

Walker. Strategies for streamlining uterine topographic classification in placenta accreta spectrum. AJOG Glob Rep 2026.

**Figure 2:**
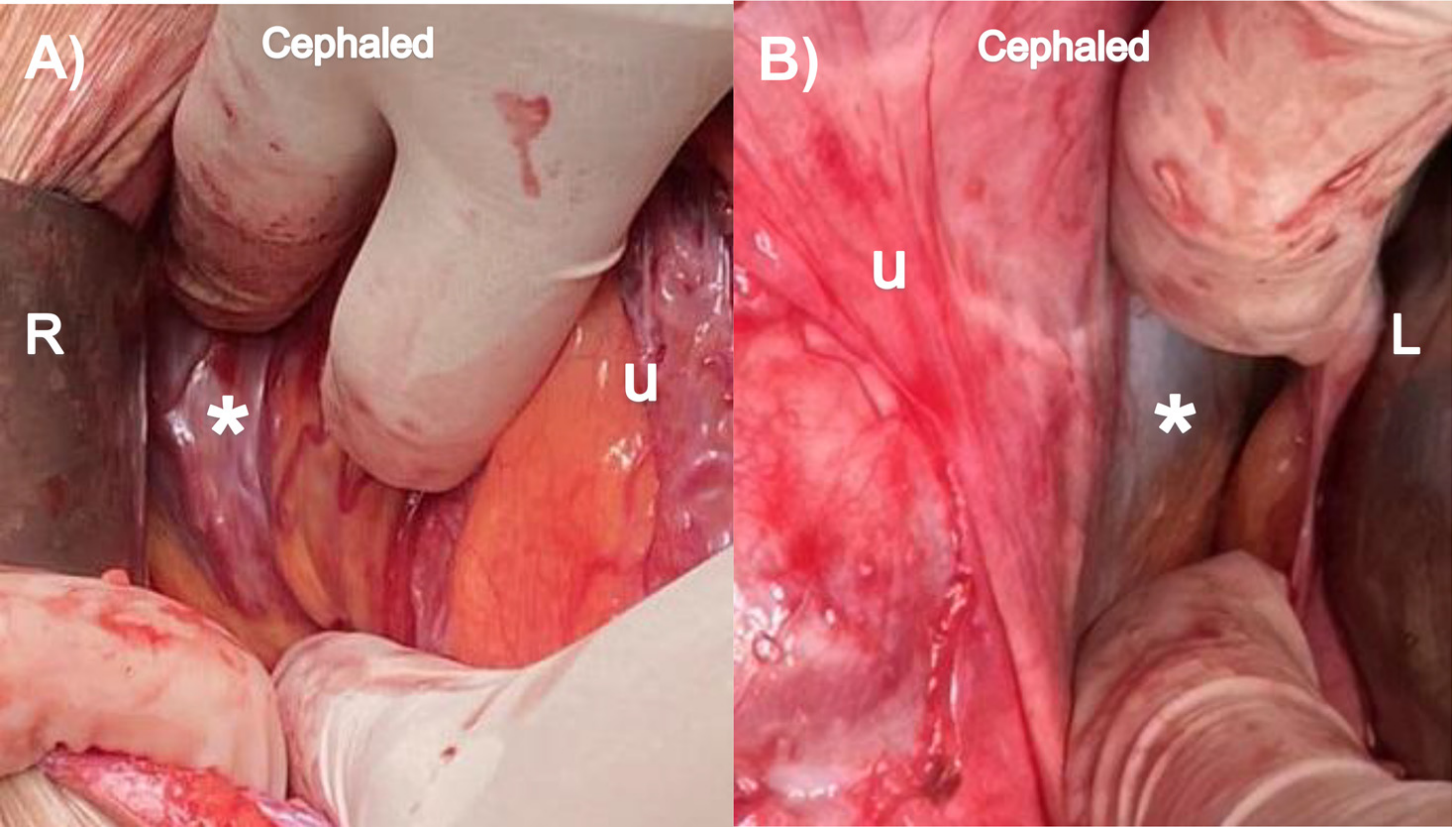
Surgical photos of the normal and abnormal appearances on opening of the parametrial space to assess lateral wall involvement (Type 2 PAS) Image (A) *shows lateral wall involvement with the presence of a bulge, bluish discoloration, and neovascularization. Image (B) *shows the normal appearances with healthy white colored myometrium, minimal bulging, and little to no neovascularization. *Cephalad*, head end of patient; *L*, patient left side; *R*, patient right side; *u*, uterus.

The main modification to practice is intraoperative surgical staging to assess the uterine wall characteristics before choosing the type of surgery to be performed.^25^^,^^26^ A detailed description of how to perform the surgical staging is illustrated in Video 1. In brief, the process involves opening both parametrial spaces to identify lateral wall involvement (Type 2). As shown in Figure 2, if the myometrial area is affected, the external surface will bulge with a bluish discoloration accompanied by neovascularization. In contrast, residual myometrium appears white or pink, with minimal bulging and little to no neovascularization. Next, continue the dissection from lateral to medial to expose the anterior uterine wall, rule out the presence of dense adhesions between the lower part of the uterus and the bladder (Type 4), and evaluate the extent of anterior wall involvement (Type 1 or 3) and the presence of healthy residual myometrium cephalad to the cervix. Then, after fetal delivery, exteriorizing the uterus to look for posterior wall involvement (Type 5).^26^

Walker. Strategies for streamlining uterine topographic classification in placenta accreta spectrum. AJOG Glob Rep 2026.

**Figure 3:**
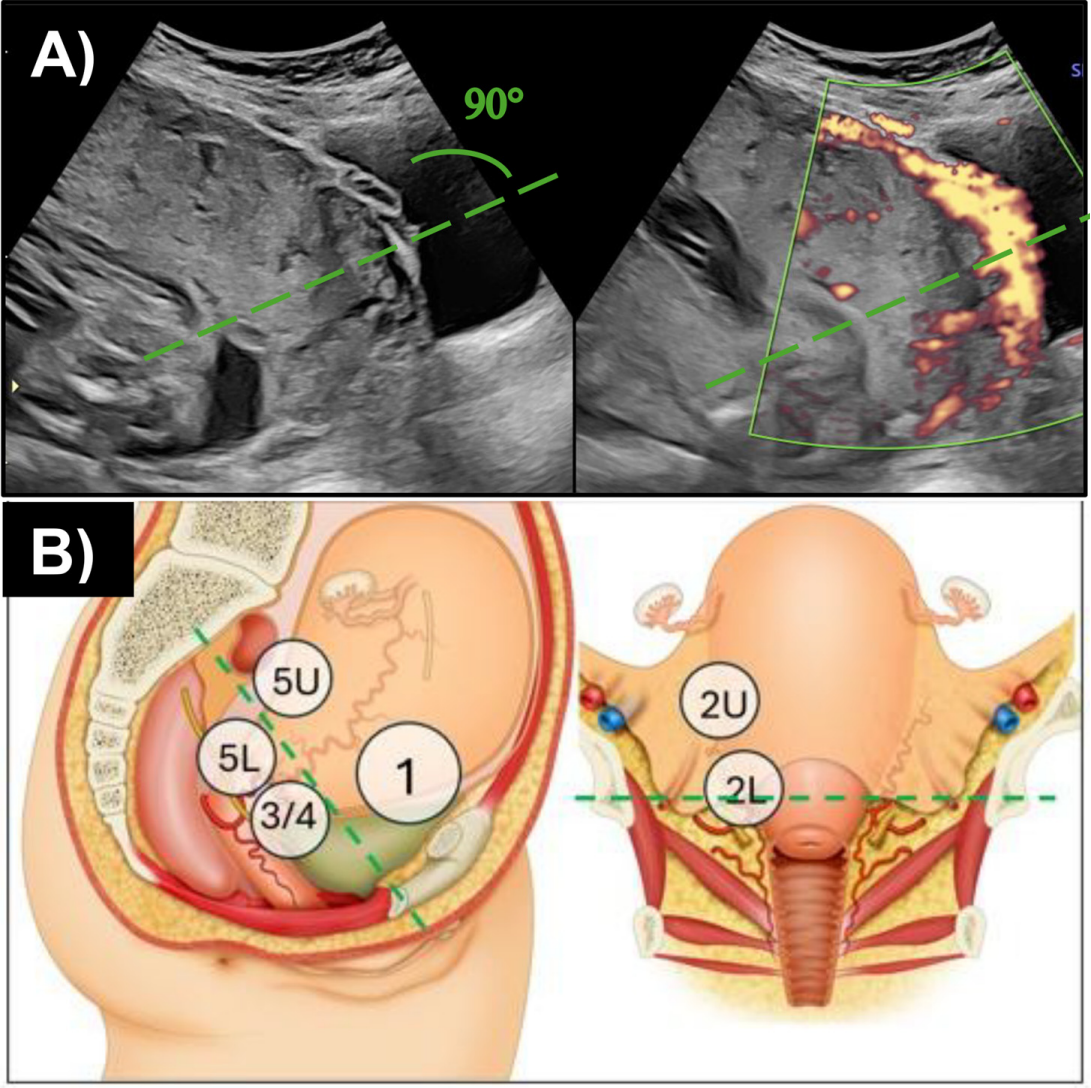
Illustration of the two topographical sectors The peritoneal reflection serves as the landmark for classifying topographic types by dividing the upper (sector S1) and lower (sector S2) parts of the LUS.^27^ The ultrasound image (A) shows how prenatally, with a full bladder, the boundary approximates with an imaginary line that perpendicularly bisects the posterior bladder wall (green line). Image (B) illustrates the sagittal view of how, intraoperatively, when the bladder is empty, the peritoneal reflection corresponds lower down, and the vesicouterine space needs to be opened to differentiate between the upper (Type 1) and lower (Type 3/4) lesions. The green line demarcates the peritoneal reflection.

The peritoneal reflection serves as a key landmark for classifying topographic types by determining whether the uterine defect lies in the upper (sector S1) or lower (sector S2) part of the LUS.^27^ In this construct, sector S1 comprises the body of the uterus, and sector S2 corresponds to the LUS and cervix.^28^ Figure 3 illustrates how these sectors are delineated. Prenatally, with a full bladder, ultrasound approximates the boundary using an imaginary line that perpendicularly bisects the posterior bladder wall. Intraoperatively, however, the peritoneal reflection is often displaced superiorly due to adhesions from prior cesareans. Therefore, opening the vesicouterine space is essential to differentiate between upper defects (Type 1), where substantial healthy myometrium exists cephalad to the cervix, allowing for OSCS, and lower defects (Type 3/4), where limited healthy myometrium above the cervix makes OSCS less feasible, except in experienced hands and when no additional complicating factors are present. The steps involved in performing OSCS are demonstrated in Video 2.

Walker. Strategies for streamlining uterine topographic classification in placenta accreta spectrum. AJOG Glob Rep 2026.

The true value of the topographical classification lies in it guiding appropriate management strategies.^1^ This classification means that defects in the lower part of the uterus (below the peritoneal reflection) are more complex to manage. Two types of defects should be ruled out first by the surgeon due to their severity: (1) Low lateral (Type 2L) due to their close connection with the large pelvic vessels and ureter, and the narrowness of the space in which they are located. (2) Low anterior defects, in which it is impossible to separate the bladder from the uterus (Type 4), making massive bleeding (with 93.7% of cases requiring blood transfusion) and urinary tract injuries common (46.9% bladder injury and 6.2% ureteric injury).^29^ In these two types of lesions, management should deviate from the usual, and the team should utilize all available resources to improve outcomes. Once these situations have been ruled out, the numbering of each category is secondary. Although posterior injuries are designated Type 5, lateral injuries Type 2, and anterior injuries Type 1 or 3, describing the topography of the abnormal myometrium by the affected uterine wall is equally appropriate.

Once the extent of the uterine wall involvement is determined, the appropriate vascular control strategy and surgical treatment, as outlined in Table, can be decided. Notably, some defects will be diffuse, encompassing more than one area, such as a Type 1 PAS involving more than 50% of the uterine circumference, making it synchronized with Type 2U. In this case, uterine conservation is not feasible due to the extensive defect.^24^^,^^26^ In Type 3 cases, at least 2 cm of healthy lower uterine tissue is recommended as prerequisite to try focal resection and uterine reconstruction (OSCS).^26^ However, with advanced surgical experience, conservative surgery may still be successfully performed in selected cases with only a small amount of healthy myometrium above the cervix.

A deeper understanding of experience and its correlation with surgical staging, as highlighted by the traffic-light color coding in Table, enhances its utility in clinical decision-making.^30^

## Vasculature of the uterus

Once the topographical area of the defect is identified, the corresponding vascular supply can be determined to guide targeted devascularization strategies.^1^ The uterus is divided into two sectors, again divided by the peritoneal reflection, based on the main arterial pedicles irrigating each sector. Sector S1, corresponding to the uterine body, is predominantly supplied by the uterine artery, with additional input from the ovarian artery. Sector S2, corresponding to the LUS and cervix, receives its main blood supply from the colpouterine arteries.^31^

Sector S2 is the region most commonly affected in placenta previa, uterine dehiscence, and PAS.^31^ As such, ligation of the colpouterine arteries is critical for achieving hemostasis in uterine-sparing procedures. Vascular devascularization is not achieved by directly identifying and ligating individual arteries, but rather by compressing the anatomical regions through which these vessels run. Identifying and examining the artery is unnecessary; it is sufficient to know which area of the uterus is bleeding, remember which arterial pedicle supplies that area, and define the expected technical difficulty to achieve the surgical dissection required in each case.^32^ This anatomical knowledge helps the surgeon to recommend the vascular control strategy for each category of the topographic classification.^30^ In the case of uterine-sparing surgery, this is done by placing deep, wide hemostatic stitches with maximal tension. Persistent bleeding from the placental bed suggests that a vascular area has not been adequately compressed. In such cases, placing overlapping sutures or applying deeper and wider sutures usually results in effective hemostasis. Make sure to have identified the ureter and not place your sutures lower than the level of bladder reflection.

**Figure 4:**
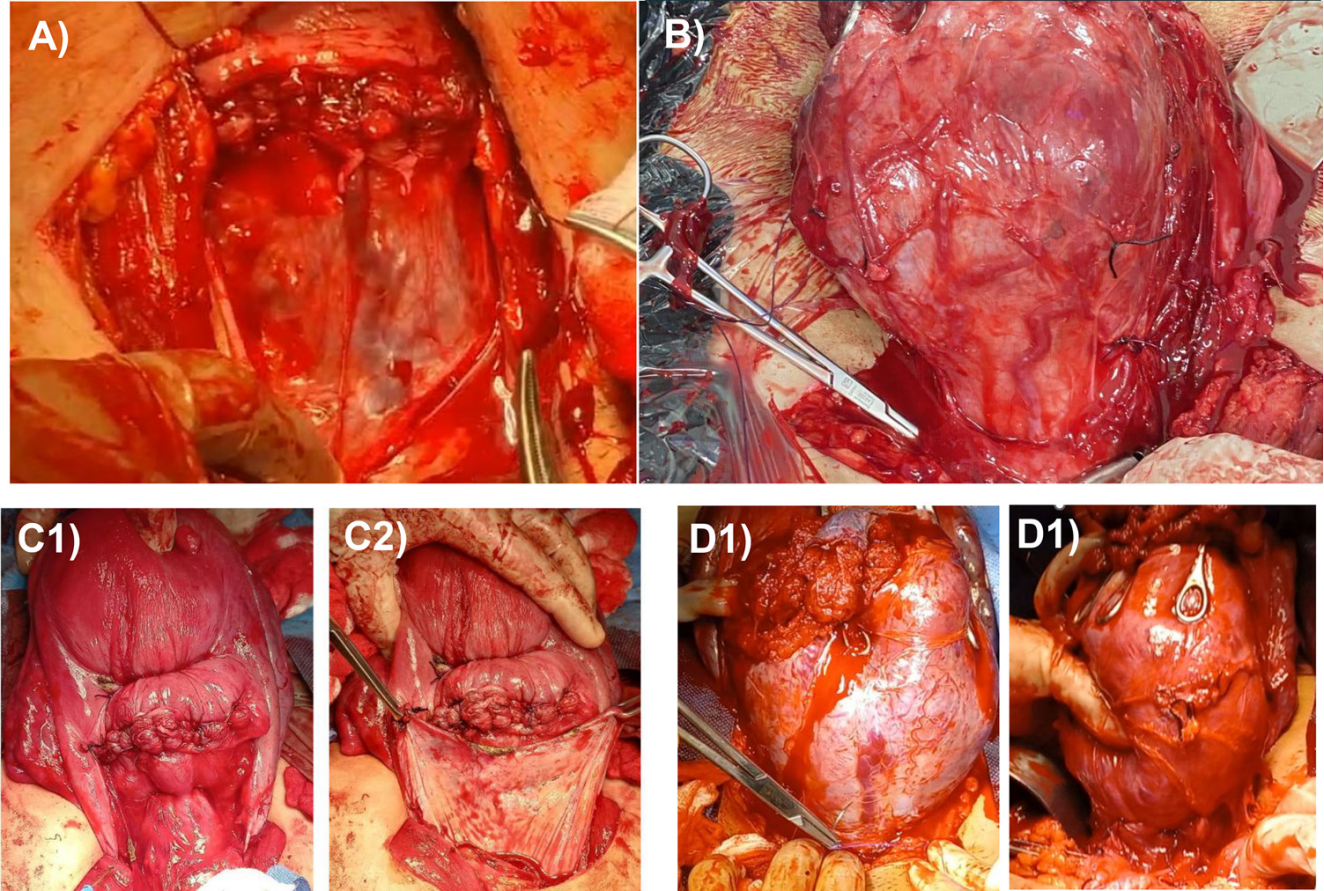
Colpouterine arteries in patients with placenta previa and placenta accreta spectrum (A) Colpouterine arteries in a patient with placenta previa (after exposing the lower uterine segment by dissecting and mobilizing the bladder). (B) Colpouterine arteries in a patient with placenta accreta spectrum. (C) Low compression suture, Type B-Lynch 2 (transverse B-Lynch), over sector 2 in a patient with postpartum bleeding following delivery of a placenta previa (C1), showing the location of the suture in a topography that is normally covered by the bladder (C2). (D) Ligation of the colpouterine arteries prior to en bloc resection in a patient with placenta accreta spectrum. Image (D1) shows the moment before ligating the colpouterine artery in the midline (azigosvaginal artery), and Image (D2) shows the uterus with the 3 colpouterine arteries ligated, just before en bloc resection of the area affected by placenta accreta spectrum.

In cases of placenta previa, where postpartum bleeding after placental separation may be due to atony of the LUS where the placenta was implanted, a transverse compression suture over the LUS (including the colpouterine vessels) is almost always sufficient, without the need to place hemostatic stitches specifically targeting the location of these vessels (Figure 4).

Walker. Strategies for streamlining uterine topographic classification in placenta accreta spectrum. AJOG Glob Rep 2026.

## Intracervical hypervascularity

**Figure 5:**
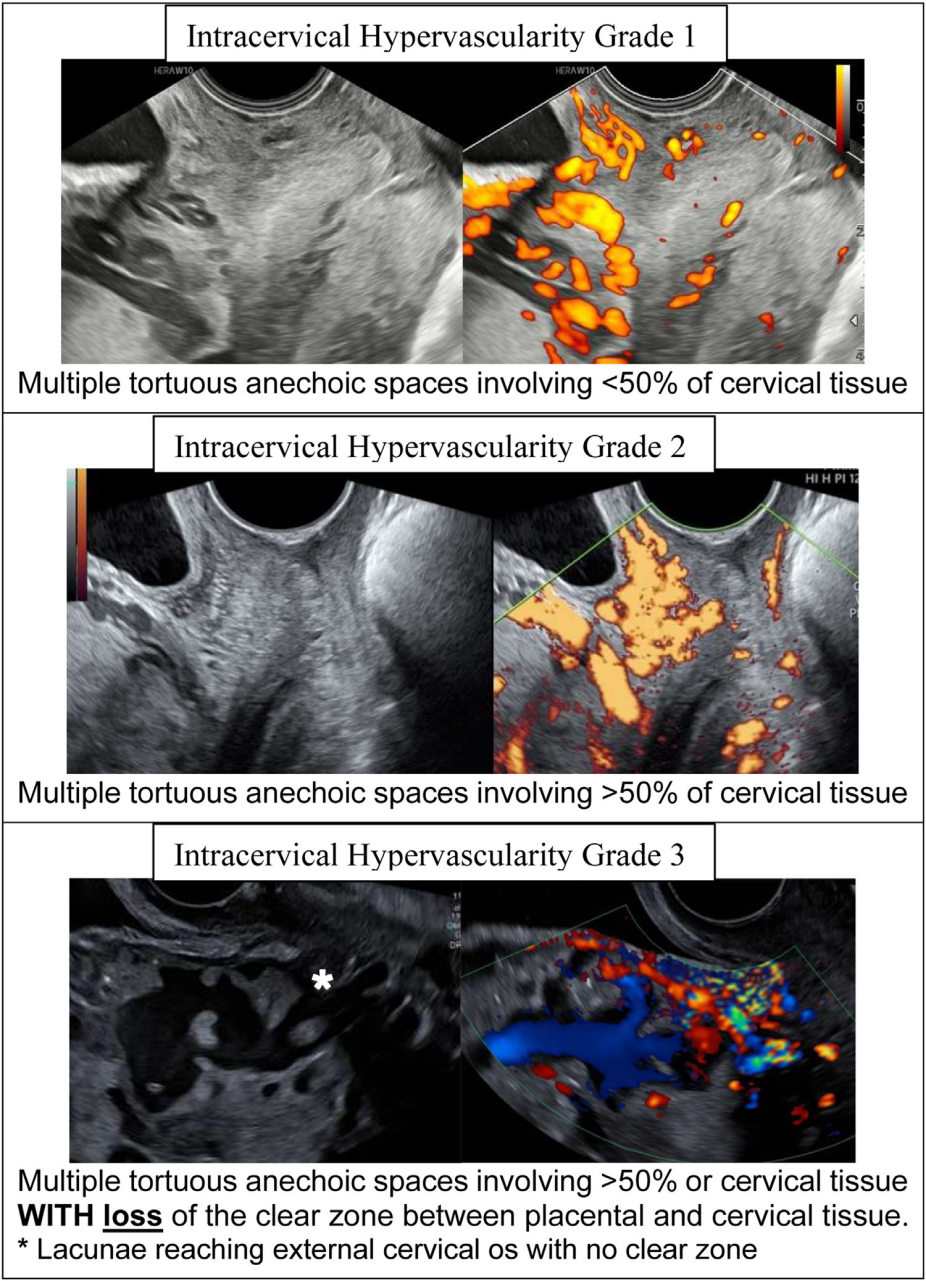
Intracervical Hypervascularity (ICH) Grading System Aryananda et al,^32^ propose a three-level grading system based on the extent of multiple tortuous anechoic spaces within the cervix.

Intracervical hypervascularity or intracervical lakes are independently associated with an increased risk of major PPH.^33^^,^^34^ Figure 5 outlines a recently proposed grading system. The more vascular the cervix appears, the less success in uterine-sparing surgery.^33^

Walker. Strategies for streamlining uterine topographic classification in placenta accreta spectrum. AJOG Glob Rep 2026.

Grade 2 and 3 intracervical hypervascularity are rarely seen in upper topographical defects. When grade 3 is present, OSCS will not succeed and should be avoided.^34^^,^^35^ The primary maternal risk in these cases is severe, rapid vaginal bleeding during surgery.^36^^,^^37^ Accurate prenatal identification of this ultrasound finding is critical, allowing for planned caesarean hysterectomy without placental disruption to reduce vaginal blood loss.^36^ When encountered, internal manual aortic compression offers a rapid, simple, and effective method to assist in hemorrhage control.^38^ As with other strategies for aortic occlusion (cross-clamping or intra-aortic balloon), if available.

## Topographical cases encountered in low-volume PAS referral centers

Estimates of the distribution of topographical PAS types have been derived from data reported by two high-volume referral centers that incorporate topographical grading into their clinical management. A tertiary referral center in Indonesia recently published its annual caseload from September 2023 to August 2024, comprising 123 cases: 30 Type 0 (24.4%), 63 Type 1 (51.2%), 5 Type 2 (4.1%), 17 Type 3 (13.8%), and 8 Type 4 (6.5%).^35^ Similarly, a large tertiary referral center in Argentina reported outcomes from 452 cases, of which 126 were Type 0 (27.9%), 248 Type 1 (54.9%), 44 Type 2 (9.7%), 23 Type 3 (5.1%), and 11 Type 4 (2.4%).^11^

Therefore, it can be extrapolated that the most common PAS cases encountered are PAS Type 0 and 1.^11^^,^^35^ Effective management hinges on developing a systematic approach to prenatally define characteristics of each patient and establish a surgical plan, including options if complications arise. All this added to meticulous dissection for safe and effective treatment. Over time, clinicians will become more competent at performing successful OSCS, lowering hysterectomy rate, reducing bladder injuries, and reducing blood loss. Gaining initial experience with surgical staging can be challenging, as it requires additional expertise in bladder dissection and colpouterine control. Understanding the danger signs on prenatal ultrasound, such as advanced grading of intracervical hypervascularity, might help as a guidance for using prophylactic abdominal aortic control to prevent unexpected massive bleeding during surgical staging.^30^^,^^34^

Type 4 PAS is rare and may typically be encountered in low-volume PAS referral centers no more than once every 1 to 2 years. It is characterized by extensive fibrosis, which renders surgical dissection particularly complex. MSTH has been associated with lower perioperative morbidity.^29^^,^^38^ Type 2L PAS is also extremely rare, and its surgical complexity stems from the involvement of many arterial pedicles originating from multiple sources, which obstetricians are not frequently required to ligate.^39^ Furthermore, the proximity of the ureter and associated neovascularization from the ureteral artery necessitates precise ureteral dissection.^39^

In the complex cases (Type 2L, Type 4, and Type 5L), infrarenal aortic vascular control is recommended to help control pelvic bleeding.^30^^,^^38^ In Type 2L cases, ureteral stents can assist the surgeon in identifying the ureter, which is displaced by the lateral bulging caused by involvement of the lateral uterine wall. A rational approach for optimizing outcomes is to centralize the management of such complex cases within a limited number of high-volume national referral centers. This approach not only ensures access to experienced multidisciplinary teams but also provides the optimal environment for training the next generation of specialists. With greater exposure to the full spectrum of PAS complexity, these surgeons can subsequently extend their expertise to lower-volume centers, thereby enhancing local capacity for managing less complex cases. By contrast, if all hospitals attempt to manage PAS cases independently, surgical expertise becomes diluted, reducing opportunities for both training and surgical improvement. A recent United Kingdom survey revealed that many cases continue to be managed in centers not formally recognized as specialist units, despite growing evidence that morbidity is significantly lower when patients are referred to high-volume centers with greater expertise.^40^

## Training and collaboration

While no definitive case-volume threshold exists, maintaining proficiency in PAS surgery likely requires a multidisciplinary team managing several cases per year, with structured approaches including joint surgical planning, recording of surgeries, outcome auditing (through video review by both the local surgical team and external peers), and continuous identification of improvement opportunities. Incorporating the practice of subperitoneal space dissection in patients without PAS but with myometrial defects and adhesions from multiple prior caesarean deliveries, as well as analyzing cases from other centers through virtual joint learning sessions, are useful strategies to achieve and maintain surgical proficiency.

Gradual implementation of new surgical techniques requires the availability of a clear plan for managing potential complications. Surgeons must anticipate scenarios such as massive hemorrhage during an attempted conservative approach and consider whether achieving en-bloc focal resection justifies the risk of severe bleeding. These considerations highlight the need for individualizing PAS management.

A critical determinant of safety during the development of surgical expertise is meticulous bladder dissection. Employing a common initial surgical approach for both conservative management and hysterectomy facilitate intraoperative decision-making by the multidisciplinary team. This is the rationale for intraoperative staging, performed before fetal extraction or the onset of bleeding. Once the topography of the lesion is identified and operative risks have been assessed, the fetus is then delivered, and any of the available surgical options can be performed. However, if adequate bladder dissection to expose the diseased area cannot be achieved, or the placental capsule is breached during dissection, it may be safer to convert to a fundal uterine incision away from the placenta and proceed with hysterectomy rather than persist with conservative surgery. With growing experience, surgeons will increasingly be able to achieve safe bladder dissection, and when the diseased area is fully exposed with healthy myometrium cephalad to the cervix, resection-reconstructive surgery can be performed safely.

It is also important that surgeons remain cautious: gaining experience with managing more routine Type 0 and Type 1 cases can build confidence, but this can be quickly challenged by complex cases that demonstrate the unpredictable and demanding nature of PAS. Practical strategies for addressing such obstacles, including escalation thresholds and decision points, are detailed in Supplemental SI.

Collaboration between hospitals is crucial. For rare and complex cases, openness to guidance from more experienced centers, combined with humility and a willingness to learn are key to improving outcomes. Visiting other centers of excellence in PAS care can provide valuable hands-on exposure and observation to alternative techniques. Telecompanionship has emerged as a valuable tool in PAS care, providing real-time remote clinical support, through secure video links or tele-mentoring.^41^ This allows experienced clinicians to guide decision-making during unexpected technical or hemorrhagic challenges, within agreed governance frameworks including institutional approval, patient consent, adherence to data security, and clear delineation of clinical responsibility. Beyond clinical support, tele-education and telecompanionship have facilitated the implementation of OSCS.^25^ For example, a prospective observational study of a virtual training program involving three expert groups and PAS referral hospitals in South America and Indonesia, centers with no prior OSCS experience, demonstrated the feasibility of such collaborative approaches.^25^ Similarly, a Central American group, through an inter-institutional collaboration program facilitated the introduction of OSCS where it had not previously been performed, and was associated with improved clinical outcomes, including fewer hysterectomy rates without an increase in urinary tract injury.^42^ In Switzerland, a low-volume national PAS referral center incorporated the topographic classification of PAS and, through a period of virtual collaboration with experts that included joint ultrasound-based surgical planning and, intraoperative telecompanionship, successfully adopted OSCS as part of their management options.^43^

However, ethical restrictions on sharing patient information and surgical images remain a major barrier in many regions. Overcoming these limitations or alternatively establishing national expert groups to provide structured support to referral centers would be highly valuable in strengthening capacity and improving care pathways.

## Should PAS diagnosis mean hysterectomy?

A rigid, tunnel-vision approach to the management of PAS, particularly in Type 0 or Type 1 cases, can be dangerous and may increase maternal morbidity or result in overtreatment. While uterine preserving techniques should be considered where appropriate, it is essential for the surgeon to recognize when these methods are not appropriate and to be prepared to transition to a hysterectomy in a timely manner. In lower-volume centers, defaulting to hysterectomy may remain the safest approach, particularly where the surgical team has limited experience or resources. At the same time, adopting structured principles, assessing lesion type, anticipating surgical complexity, and gradually building confidence in conservative approaches is important. With adequate surgical training and a strong understanding of anatomical and vascular principles, bleeding in many Type 0 and 1 PAS cases can often be effectively controlled without resorting to hysterectomy.^11^

It is also important to recognize that uterine dehiscence can be complex and may present challenges equal to or greater than some PAS cases. Centers developing expertise in both conditions, supported by structured training and multidisciplinary collaboration, are likely to achieve the best maternal outcomes. In this context, hysterectomy should remain an available and safe option, but its use should be guided by careful assessment rather than reflexive practice.

It is important that clinicians avoid misclassifying cases as PAS to retrospectively justify surgical outcomes. Such false-positive diagnosis limits our collective understanding of the etiology, clinical behavior, and optimal management of these cases.^20^^,^^21^ Furthermore, it is essential to provide thorough preoperative counselling discussing the risks and benefits of available management strategies while incorporating the patient’s preferences into decision-making.

## Conclusion

A clear understanding of uterine topography and vascular anatomy is fundamental to the effective management of PAS, uterine dehiscence, and anterior placenta previa. By presenting a simplified, practical framework based on these principles, the article aims to support clinicians working in PAS referral centers that manage relatively few cases each year, facilitating safer and more individualized surgical decision-making.

Recognizing anatomical patterns, anticipating complications based on topographical type, and tailoring vascular control strategies to each case allows for more confident, conservative surgical approaches when appropriate, and timely transition to hysterectomy when required.

Although, most cases of PAS will fall within the more manageable upper peritoneal reflection PAS types, clinicians must be prepared for the occasional complex or rare presentation. In these ongoing collaboration between institutions, humility in seeking support, and integration of telecompanionship can help ensure that these rare cases are handled with the benefit of collective expertise.

## Declaration of AI and AI-assisted technologies in the writing process

There was no use of AI or AI-assisted technologies in the writing of this manuscript.

## CRediT authorship contribution statement

**Sarah P. Walker:** Writing – original draft, Visualization, Conceptualization. **Helena C. Bartels:** Writing – review & editing, Visualization, Conceptualization. **Albaro J. Nieto-Calvache:** Writing – review & editing, Visualization, Validation. **José M. Palacios-Jaraquemada:** Writing – review & editing, Validation. **Sally L. Collins:** Validation. **Rozi Aryananda:** Writing – review & editing, Supervision, Conceptualization.

## Appendix Supplementary materials

Image, application 1

Image, application 2

Image, application 3

## Footnotes

Supplementary material associated with this article can be found in the online version at doi:10.1016/j.xagr.2026.100636.
