# Supplementary material for: Strategies for streamlining uterine topographic classification in placenta accreta spectrum
Source: AJOG Glob Rep. 2026 Mar 26;6(2):100636. doi: 10.1016/j.xagr.2026.100636 (PMC13126007; doi:10.1016/j.xagr.2026.100636)
Supplement: Supplementary file 1 [file mmc1.docx]

| **Obstacles Encountered** | **Strategies to Overcome Obstacles** | | | | |
| --- | --- | --- | --- | --- | --- |
| **Damage to placental capsule prior to completion of bladder mobilisation** | 1. **Avoid clamping the tissue**, as it is extremely fragile and may tear further 2. **Apply direct pressure** using a swab or finger over the bleeding point 3. **If identifiable and small bleeding point**, consider ligation of the colpouterine artery below the bleeding site 4. If space allows, attempt to clamp the edges of the rupture area with an atraumatic vascular clamp. 5. **If bleeding not controlled**, discontinue bladder dissection and proceed to deliver the baby. After delivery, exteriorize the uterus and perform colpouterine artery ligation to achieve hemostasis. 6. If initial plan had been OSCS, have a **low threshold** to transition to perform a hysterectomy if blood loss is higher than anticipated and unable to control by other methods 7. Consider **IMAC** for immediate control and to gain time. | | 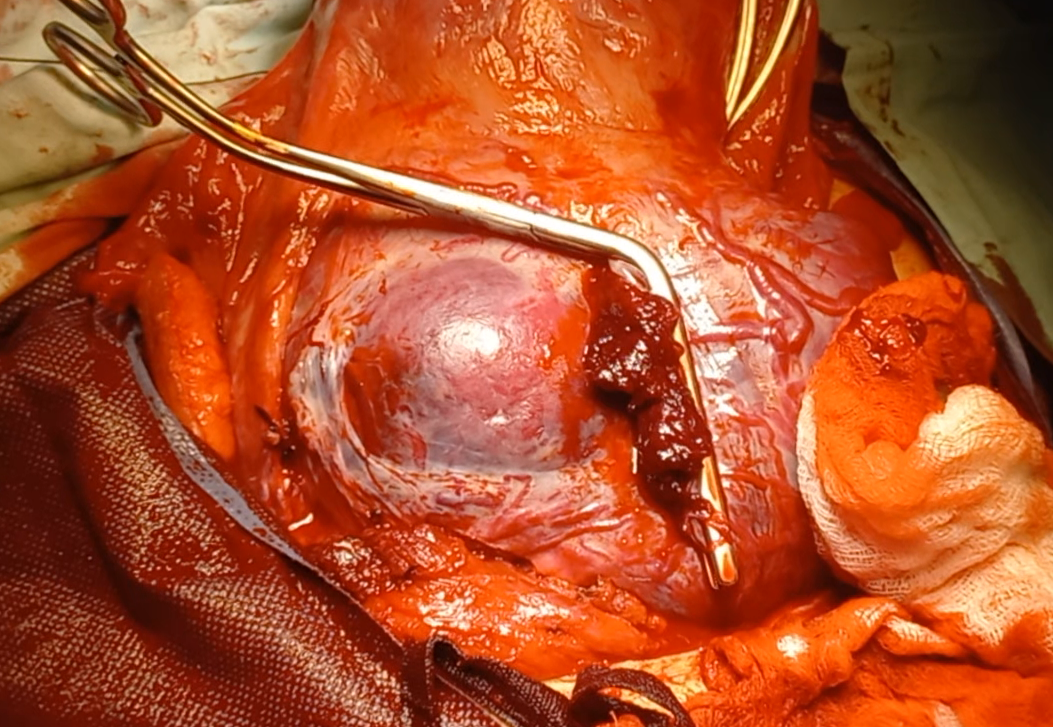  Image of a vascular clamp compressing the bleeding edges of a capsule rupture in the area affected by accreta and allowing bladder dissection to continue without active bleeding. | | |
| **Bladder Injury** | 1. **Identification of injury is crucial**. If uncertain, fill the bladder with dye to confirm the location. If cell salvage is available, use **patent blue** rather than **methylene blue**, as it allows for continued use of salvaged blood. 2. **Call Urology** for assistance if needed. | | | | |
| **Cannot continue bladder dissection as too much fibrosis** | 1. If feasible, locate and ligate the **vesicouterine vessels.** The vesicouterine vessels contribute blood supply to the central lower anterior uterine wall - typically the location of the hysterotomy (Sector S2). Pre-emptive ligation of these vessels can significantly reduce blood loss following hysterotomy. 2. Vesicouterine adherence can occur in the upper posterior bladder, or throughout the entire posterior bladder. 3. For upper posterior vesicouterine adherence, consider using **Pelosi technique** to create a retrovesical tunnel. Then pass a Satinsky vascular clamp through the tunnel, under the area of bladder adherence to occlude the vesicouterine vessels within that area before proceeding with delivery. 4. If bladder dissection remains unachievable in the lower uterine segment, even after delivery and exteriorisation of the uterus- as in cases of Type 4 PAS- consider proceeding with a MSTH. | 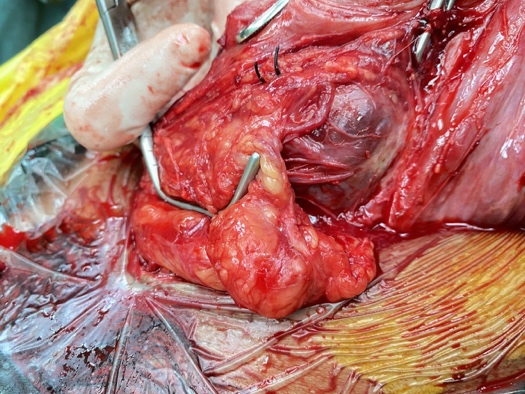  b  u  Image of the Satinsky clamp (white arrow) passed through the created retrovesical tunnel, occluding the vesicouterine vessels within the upper part of the posterior bladder (b) adherent to the uterus (u). | | | |
| **Where’s the Ureter?!** | 1. **The uterosacral ligament** serves as a key anatomical landmark for avoiding the ureter. The ureter runs along the lateral side of the uterosacral ligament and enters the endopelvic fascia before coursing anteromedially toward the bladder trigone (26). Therefore, maintaining suture placement superior to the uterosacral ligament- especially when lateral- avoids ureteral entrapment. 2. **Avoid opening the retroperitoneum** unless necessary. In most cases, it is not required and may provoke bleeding due to the highly neovascularized tissues commonly seen in PAS. 3. Opening the **medial paravesical spaces** mobilizes the ureters laterally and reduces the risk of injury during dissection. 4. **Limiting dissection** to the level where healthy myometrial tissue is observed (in type 1 and some type 3 lesions), and performing one-step conservative surgery when appropriate, reduces the need to operate near the ureter (to complete a hysterectomy) in most patients. 5. **Ureteric Stents** can be beneficial in lower topographical types, particularly in Type 2L, by facilitating intraoperative identification. 6. **Training in ureter identification** during complex cesarean deliveries without PAS (a procedure far more common than PAS surgery in low-volume hospitals) facilitates ureteral identification in more severe cases | 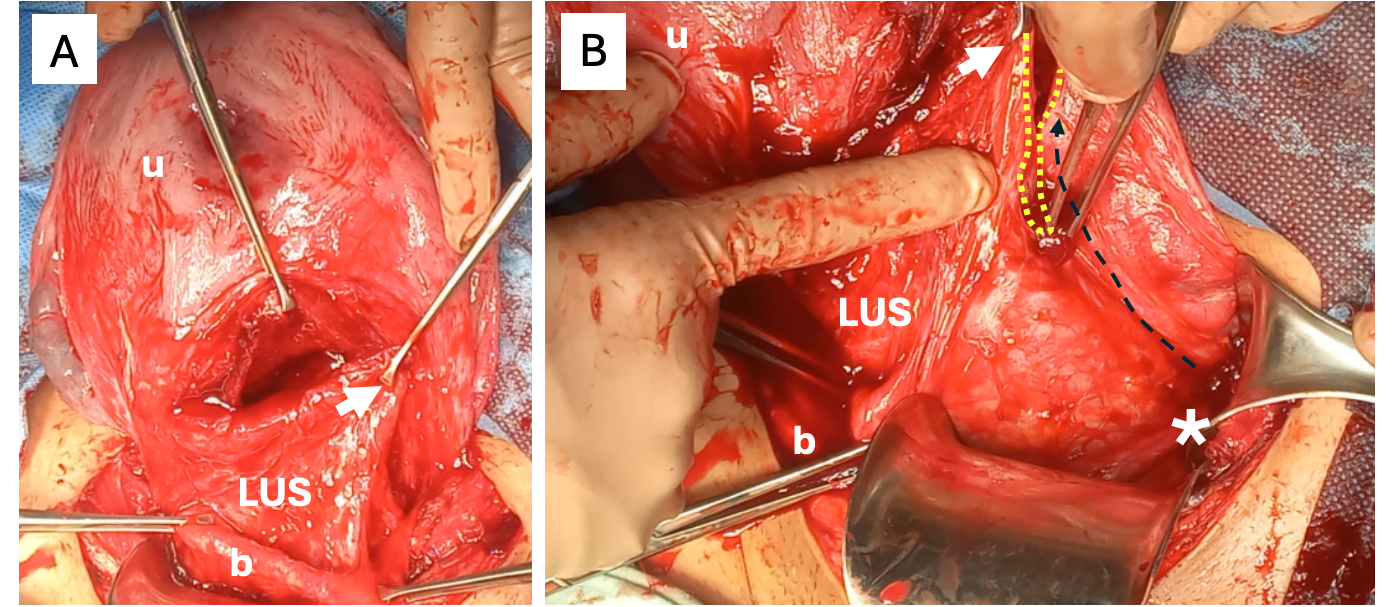  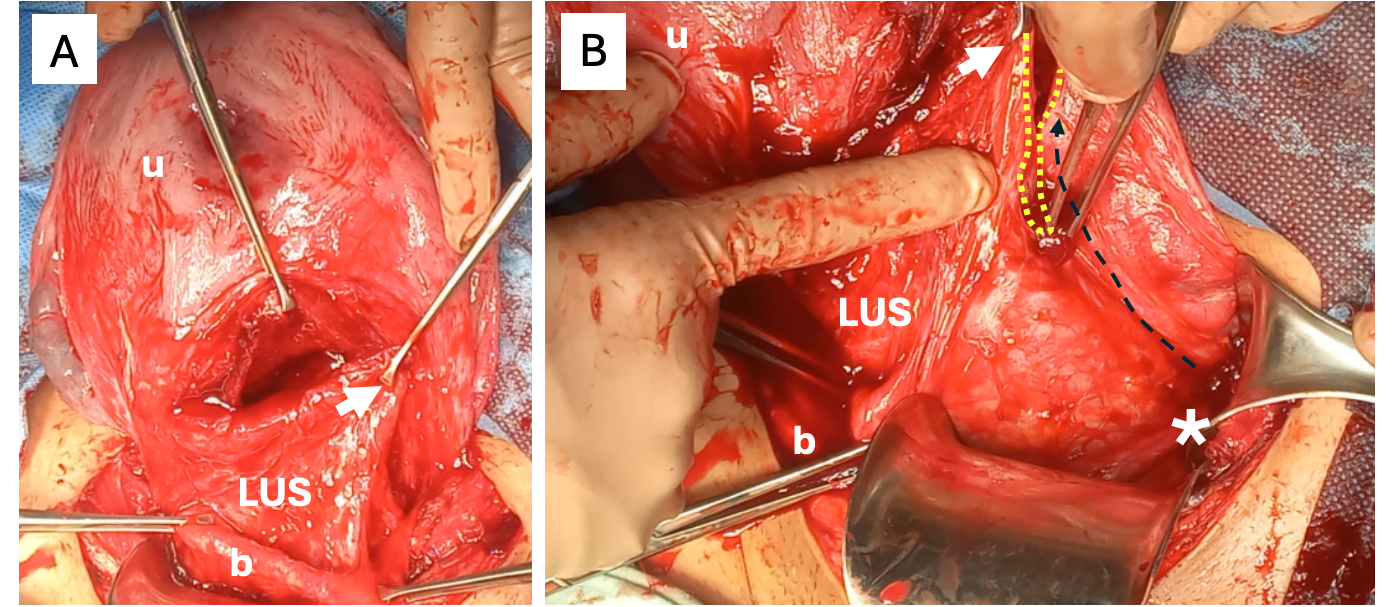  Relationship between the ureter and the LUS in a patient with lateral extension of the hysterotomy.  A. Bladder dissection (b) has been initiated to expose the LUS. The angle of the hysterotomy (arrow) is held with an Allis clamp, and the lateral and caudal extension is evident. B. The ureter (*) is visible along its course toward the bladder (b), in close relation to the uterine artery (black dotted line) and caudal to the lateral and downward tear (yellow dotted line) originating from the hysterotomy angle (arrow). u: uterine body | | | |
| **Protect the bowel** | 1. Careful to avoid bowel injury when applying deep sutures, especially when lateral. Care should be taken to protect the bowel, ideally with an instrument such as a **ribbon retractor**. | 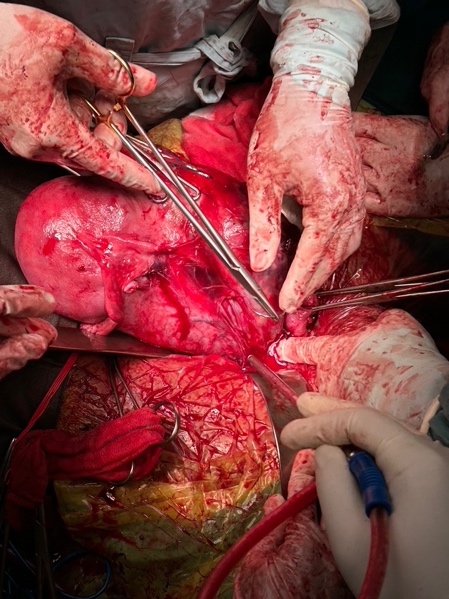 | | | Ribbon retractor (white arrow) placed behind the uterus to protect the bowel whilst a colpouterine suture is being placed laterally. |
| **Bleeding from uterine bed post colpouterine ligation** | 1. **Before proceeding with en-bloc focal resection in OSCS**, check that hemostasis has been adequately achieved via the initial ligation sutures. This can be assessed by releasing the ring clamps on the hysterotomy edge to observe the source of any bleeding. Alternatively, inspect each uterine wall individually following focal resection and placental removal. 2. Bleeding location can guide targeted hemostatic ligation: Lateral bleeding requires placement of a lateral suture to compress the uterine artery; anterior wall bleeding indicates the need for additional colpouterine sutures; posterior bleeding may require placement of hemostatic sutures to the posterior uterine wall. 3. The **“Stab test”** – a scalpel puncture at the proposed resection site- should result in minimal bleeding if vascular control is adequate. 4. To **localize bleeding** to the anterior or posterior wall, use a swab to sequentially occlude each side and observe which area reveals ongoing bleeding. | 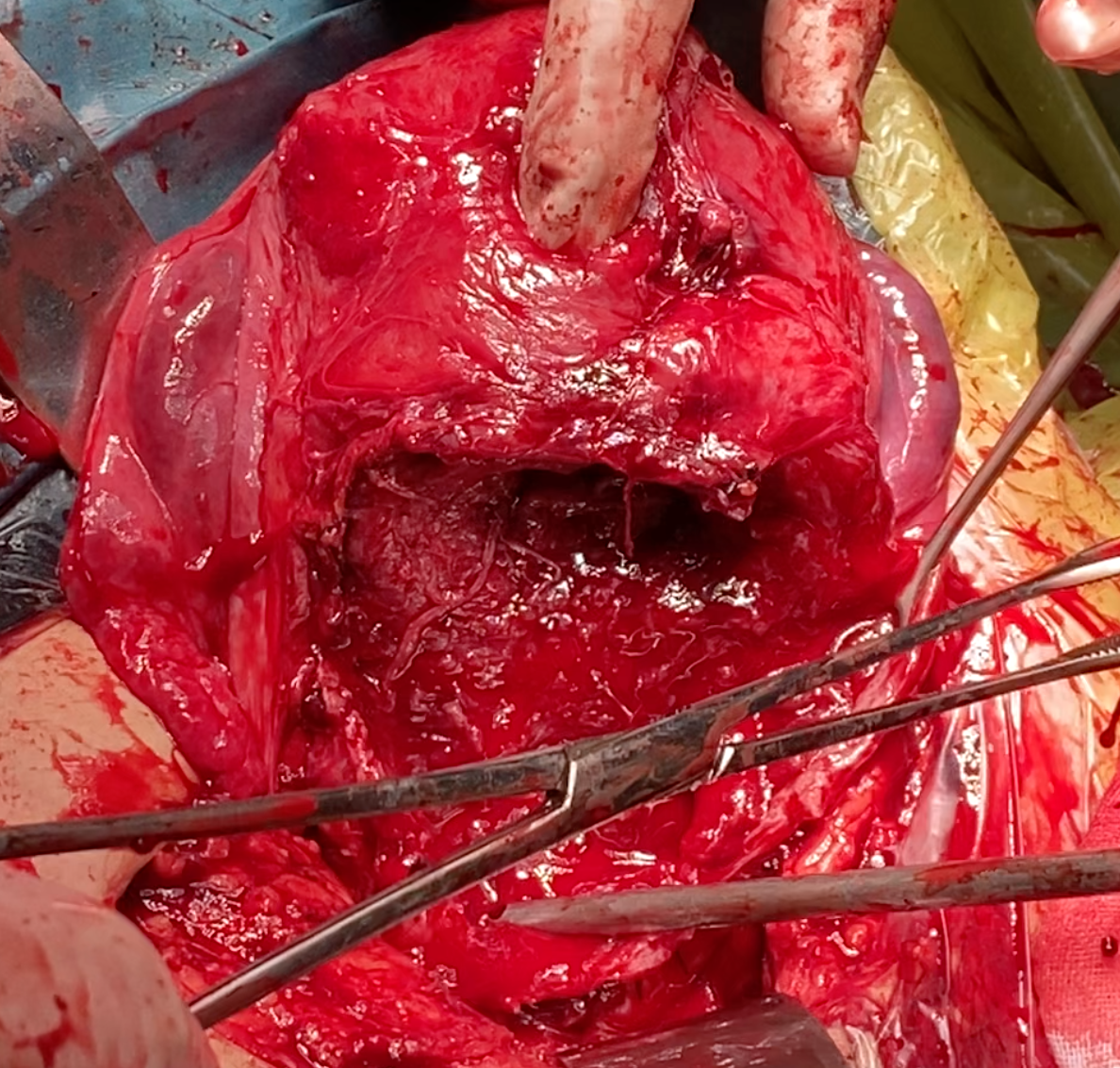  *  Image shows minimal bleeding to placental bed of hysterotomy site (*) following colpouterine ligation sutures showing adequate ligation achieved. | | | |
| **Opening space of Retzius** | This is a useful technique on opening the abdomen, when faced with dense adhesions. Opening this space allows you to feel the foley catheter balloon in the bladder and help to locate the level of the bladder. | 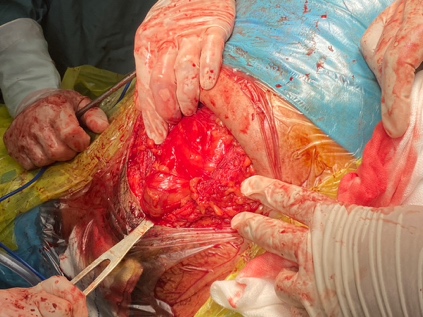 | | The space of retzius has been opened, and the arrow shows the foley catheter balloon inside the bladder. | |
| **Massive hemorrhage +/- hemodynamic instability** | 1. If have **REBOA** in situ inflate 2. Utilise **IMAC** for immediate control. * 3. Call **vascular team** – could put vascular clamp on aorta or insert REBOA if available. 4. If available prompt use of **point-of-care** coagulation monitoring and blood gas analysis, with prompt treatment of coagulopathy. 5. Have **MSTH** as a quick solution for cases which retrovesical dissection is difficult, bleeding is abundant, and the situation is urgent | 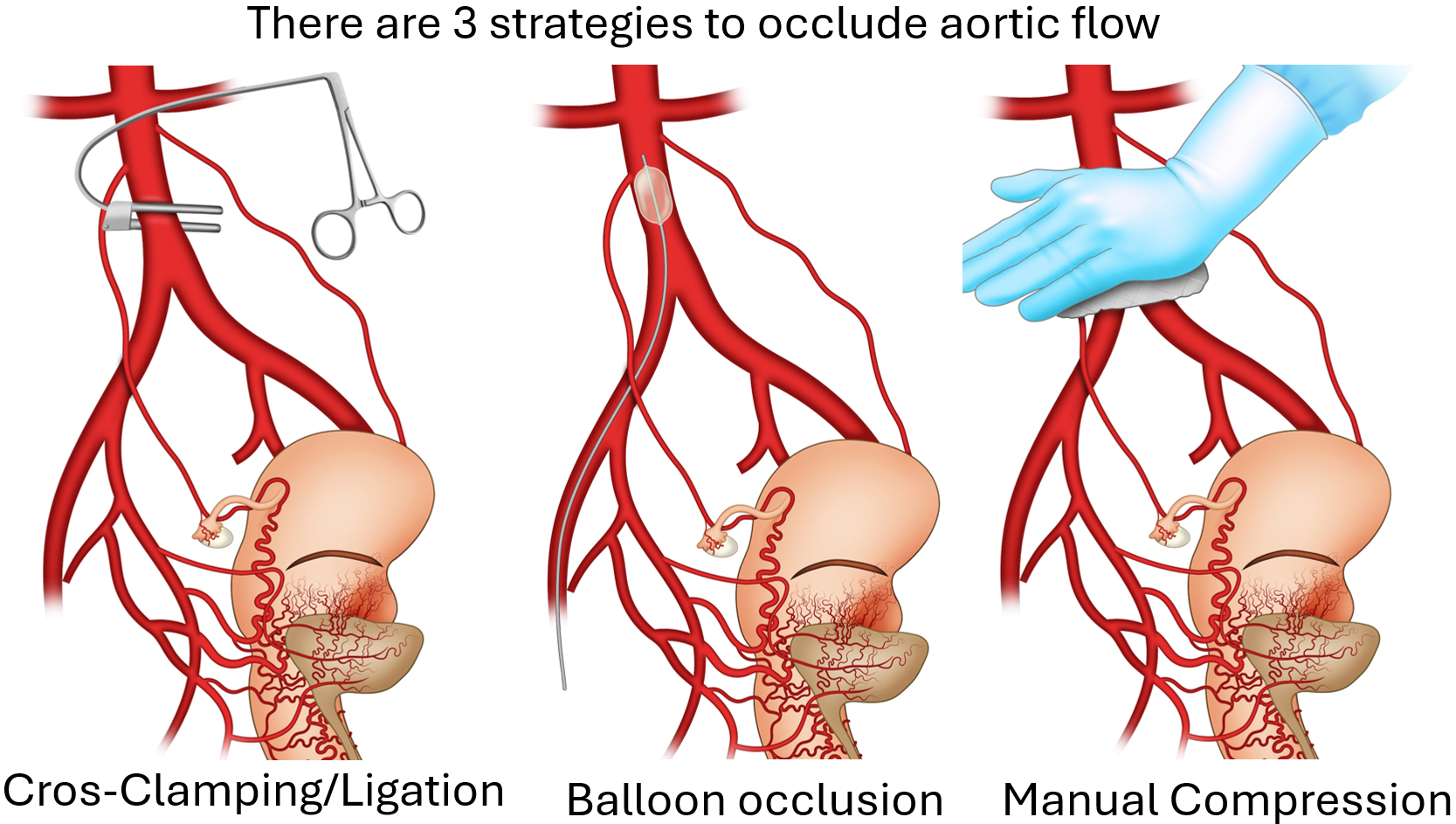  *This article describes how to perform IMAC: Nieto-Calvache, Albaro José et al. “Internal manual compression of the aorta-an effective way to temporarily control pelvic bleeding in obstetrical hemorrhage.” *American journal of obstetrics and gynecology* vol. 227,1 (2022): 96-97. doi:10.1016/j.ajog.2022.02.040 | | | |
| In all cases, if unexpected challenges arise, consider using **telecompanionship** to obtain real-time secondary surgical advice | | | | | |

**Table III: Table of common obstacles encountered and strategies to overcome them.**

LUS = Lower uterine segment, OSCS = one-step conservative surgery, IMAC = internal manual aortic compression, MSTH = Modified sub-total hysterectomy, PAS = placenta accreta spectrum, REBOA = resuscitative endovascular balloon occlusion of the aorta.
